# Unraveling the Neural Circuits: Techniques, Opportunities and Challenges in Epilepsy Research

**DOI:** 10.1007/s10571-024-01458-5

**Published:** 2024-03-06

**Authors:** Wenjie Xiao, Peile Li, Fujiao Kong, Jingyi Kong, Aihua Pan, Lili Long, Xiaoxin Yan, Bo Xiao, Jiaoe Gong, Lily Wan

**Affiliations:** 1 Department of Anatomy and Neurobiology, Central South University Xiangya Medical School, Changsha, Hunan Province China; 2 Department of Anesthesiology, Xiangya Hospital, Central South University, Changsha, Hunan Province China; 3 Department of Neurology, Xiangya Hospital, Central South University, Changsha, China; 4 Department of Neurology, Hunan Children’s Hospital, Changsha, Hunan Province China

**Keywords:** Epilepsy, Neural circuits, Techniques, Development history, Drug therapy

## Abstract

Epilepsy, a prevalent neurological disorder characterized by high morbidity, frequent recurrence, and potential drug resistance, profoundly affects millions of people globally. Understanding the microscopic mechanisms underlying seizures is crucial for effective epilepsy treatment, and a thorough understanding of the intricate neural circuits underlying epilepsy is vital for the development of targeted therapies and the enhancement of clinical outcomes. This review begins with an exploration of the historical evolution of techniques used in studying neural circuits related to epilepsy. It then provides an extensive overview of diverse techniques employed in this domain, discussing their fundamental principles, strengths, limitations, as well as their application. Additionally, the synthesis of multiple techniques to unveil the complexity of neural circuits is summarized. Finally, this review also presents targeted drug therapies associated with epileptic neural circuits. By providing a critical assessment of methodologies used in the study of epileptic neural circuits, this review seeks to enhance the understanding of these techniques, stimulate innovative approaches for unraveling epilepsy's complexities, and ultimately facilitate improved treatment and clinical translation for epilepsy.

**Graphical Abstract:**

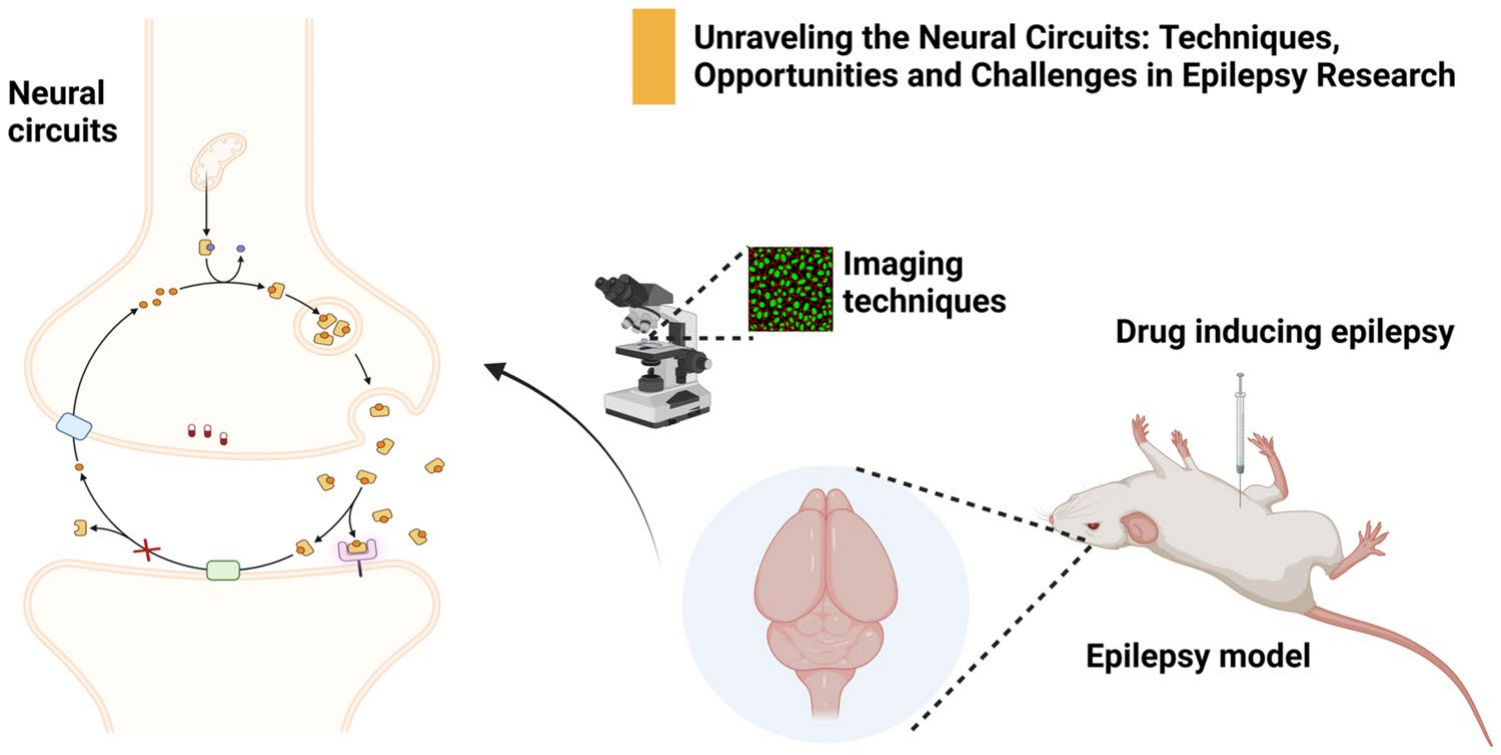

## Introduction

Epilepsy, a neurological disorder characterized by recurrent and unprovoked seizures, is a result of anomalous neural activities in the brain (Beghi et al. 2015; Beghi 2020). Globally, this disorder currently affects an estimated 65 million individuals (Akyuz et al. 2021). Understanding the microscopic mechanisms underlying seizures is crucial for effective epilepsy treatment, although the etiology of epilepsy is notably complex. It encompasses a vast array of modifications in neural circuits, which span from molecular alterations to network-level changes (Laxpati et al. 2014). Neural circuits are intricate connections formed by neurons of diverse properties and functions within the brain (Zhang et al. 2021b; Lerner et al. 2016). Neurons with distinct properties and functions establish complex connections in various forms, constituting neural circuits and neural networks at different levels (Malezieux et al. 2023). These activities take various forms, such as series, parallel, feedforward and feedback (positive or negative) (Sharpee et al. 2016). In recent years, the emergence of advanced techniques, such as viral tracing, optogenetics, and chemogenetics, has facilitated a deeper understanding of the intricate neural networks implicated in epilepsy (Xiao et al. 2022; Lv et al. 2022; Swanson et al. 2022). The application of these techniques substantially enriches our comprehension of the disorder's pathophysiology, and has been instrumental in the identification of new therapeutic targets.

The objective of this review is to provide a comprehensive summary of techniques used in studying the neural circuits associated with epilepsy, and highlight how these techniques have shaped the evolution of this research field. We also introduce the targeted drug therapy based on epileptic neural circuits to present a more comprehensive understanding.

**Fig. 1:**
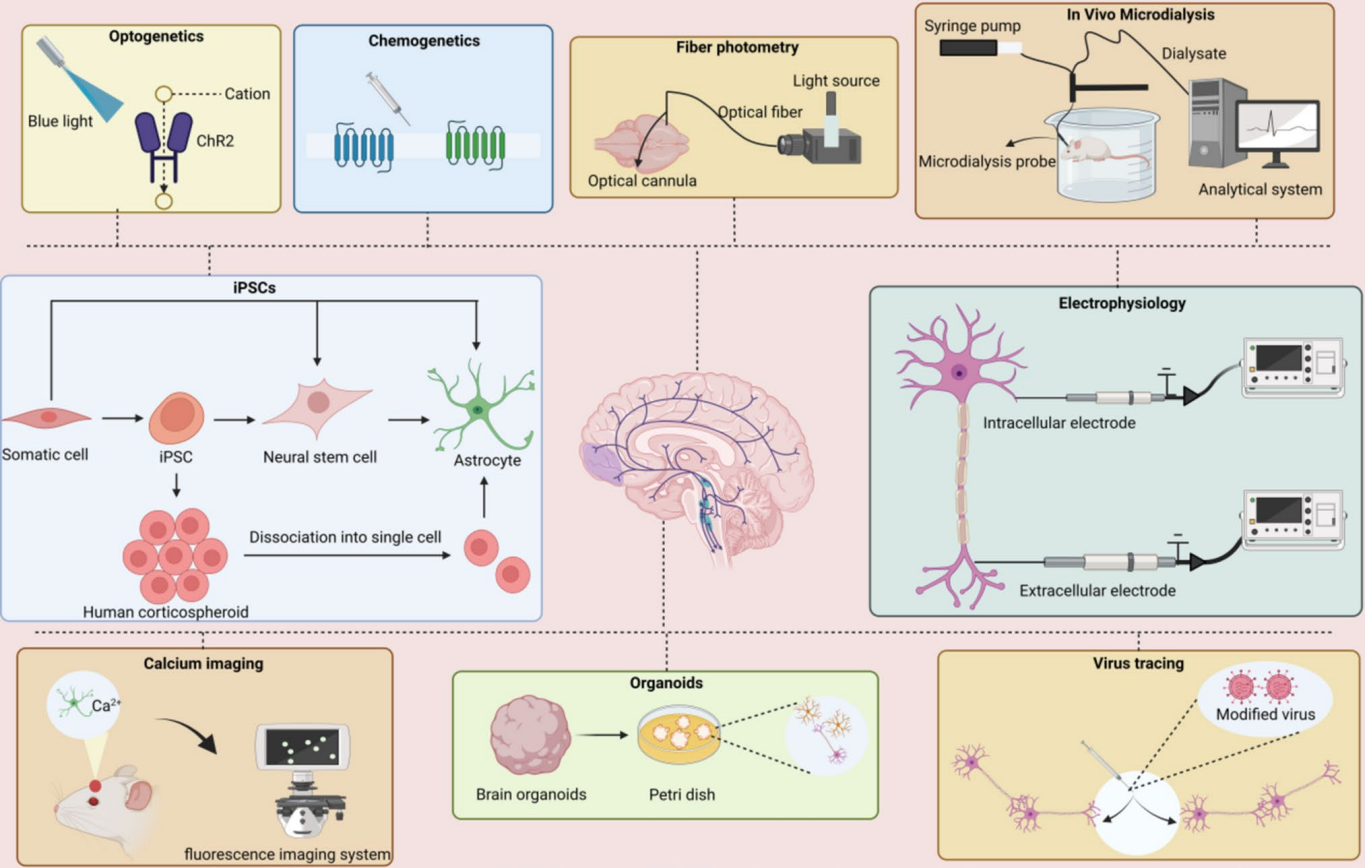
Key techniques in the study of epileptic neural circuits

This review commences by exploring the evolution of techniques used in the study of neural circuits implicated in epilepsy. Subsequently, we provide a comprehensive analysis of these methods, discussing their fundamental principles, strengths, limitations, and clinical application (Fig. 1). Key findings demonstrated from studies that have employed these techniques are introduced. Additionally, potential synergies derived from the integration of these methods are examined. We also summarize and discuss the targeted drug therapy associated with the neural circuits of epilepsy. In the end, we analyze the current findings and point out future perspectives.

### Technical Development History of Neural Circuits Researches in Epilepsy

As an ancient disorder, the history of epilepsy largely coincides with the history of human civilization. The earliest report of epilepsy dates back to about 2,000 BC (Miziak et al. 2013). For a long time in the past, there were many misunderstandings about epilepsy, which were associated with religion or culture (Ali et al. 2016). It was not until 1886 when Victor first reported on surgical treatment for post-traumatic epilepsy by performing a craniotomy, which is considered the first epilepsy surgery in history (Singh and Dua 2023; Uff et al. 2011). Psychological and behavioral methods were also applied to observe and assess epilepsy (Shafran et al. 2020; Haut et al. 2019; Cross 2015). After that, scientists began to realize that epilepsy was a neurological disorder and initiated a series of studies. With the development of imaging techniques, electroencephalography (EEG) technology, and tracing techniques, the understanding of epilepsy has greatly improved (Andrade-Machado et al. 2021; Thijs et al. 2019; Juhász and John 2020; Assenza et al. 2020). Since the discovery and proposal of neurons, generations of scientists have developed increasingly selective labeling techniques. These advancements, along with more powerful microscopic imaging techniques, have revealed neuronal morphology and projection features. Additionally, they have also discovered synaptic connections between neurons and determined the functional properties of neural circuits under these patterns of connectivity (Rubinger et al. 2017; Young et al. 2019; Hersh et al. 2022). Table 1 summarized the key advancements during technical development history of epileptic neural circuits studies.

**Table 1:** A chronicle of technologically key advancement in epileptic neural circuits research

Year	Technique	Key advancement
1846	Electrophysiology	Neural action potentials first recording
1911	Chemogenetics	Initial chemogenetic approach conceptualization
Early 1920s	Electrophysiology	Oscilloscope bioelectric recording
1970s	Optogenetics	Microbial opsins discovery and characterization
	Calcium Imaging	Inauguration of fluorescent calcium imaging
1980s	Viral Tracing	Retrograde transsynaptic tracing demonstration
	Viral Tracing	Development of attenuated PRV
1998	Chemogenetics	GPCR-based DREADDs engineering
2005	Optogenetics	Microbial opsins-mediated rapid neural activation
2006	Optogenetics	The advent of the term "optogenetics"
2007	Optogenetics	Opsins-enabled behavioral modulation
	Chemogenetics	Widespread adoption of DREADDs
	Chemogenetics	Ion channels methods inception
	In vivo microdialysis	Neurochemical monitoring in awake animal models
2009	Optogenetics	Channelrhodopsin kinetics customization
2010s	Viral Tracing	Enhancing utility via genetically modified VSV/HSV
	Fiber Photometry	Optical fibers calcium indicators recording
Recent years	Viral Tracing	Rabies virus modifications for function mapping
	Electrophysiology	Non-invasive cellular microelectrodes

The landscape of neural circuits studies has been significantly shaped by the development of innovative techniques and tools. The mainstream view is that electrophysiology was proposed by Mr. Bois-Reymond in the mid-nineteenth century (Santos et al. 2022). Afterwards, Erlanger et al. began to record bioelectricity using oscilloscopes, marking the beginning of modern electrophysiology (Breathnach and Moynihan 2014). After several generations of evolution, electrophysiology has evolved from the early stage of synchronous electrical activities of a large number of cells, to non-invasive microelectrodes at the cellular level (Fenton et al. 2022; Tomasello and Wlodkowic 2022). Calcium ions can be used as markers of neuronal excitability, and their dynamic imaging capabilities within cells have long been a focus of attention (Bonnin et al. 2024). Calcium imaging originated in the mid-1970s, proposed by Blinks et al. (Drew et al. 2019). At the same period, in vivo microdialysis has also been improved, enabling its real application in the quantitative analysis of neurotransmitter levels within awake experimental animals (Krebs-Kraft et al. 2007). After that, Gunaydin et al. proposed fiber photometry to track specific signals in axons when animals respond to stimuli (Gunaydin et al. 2014). Compared to several other technologies, the development history of techniques that are commonly applied, such as viral tracing, optogenetics, and chemogenetics, is more comprehensive.

Viral tracing has proven to be an integral technique for visualizing neural circuits. Initial studies have highlighted the importance of neurotropic viruses in mapping neural circuits, which is due to their ability to replicate within infected neurons and to label hierarchical neural circuits through self-amplification (Kuypers and Ugolini 1990; Martin and Dolivo 1983). Subsequently, the emergence of an attenuated strain of PRV demonstrated highly specific retrograde transneuronal infections (Enquist 2002; Loewy 1998). Further refinements to the Bartha PRV have resulted in a potent tool for retrograde viral tracing, lending itself to the analysis of neural circuits in non-primate species (Jia et al. 2019). Genetic modifications to the VSV and HSV have also enhanced their utility in neural circuits studies (van den Pol et al. 2009; Beier et al. 2011, 2013). In recent years, advancements in the rabies virus modification techniques have made it an invaluable tool, capable of expressing numerous essential genes to correlate circuits to function (Sun et al. 2019c; Suzuki et al. 2019).

Optogenetics and chemogenetics are two modulatable techniques with extensive applications in manipulating neural circuits. Their widespread acceptance and application in neural circuit studies have been instrumental in our current understanding of the neural circuits involved in epilepsy. The first chemogenetic approach was demonstrated in 1911, with its great potential presented (Strader et al. 1991). Then, the second-generation tools called receptors activated exclusively by synthetic ligands, engineered from native G-protein-coupled receptors, were described (Coward et al. 1998; Chang et al. 2007). Since designer receptors exclusively activated by designer drugs (DREADDs) were first pointed out in 2007, DREADDs-based approaches have been widely adopted and improved (Armbruster et al. 2007; Vardy et al. 2015). In parallel to the development of DREADDs, ion channel-based approaches were also developed (Lerchner et al. 2007).

As to optogenetics, it can be traced back to 1971, when microbial organisms were found to have the potential to produce and use rhodopsin-like proteins (Oesterhelt and Stoeckenius 1971). After that, some early approaches were developed and applied. A huge breakthrough in its application to neuroscience was the discovery that neurons could rapidly respond to light when a microbial opsin gene was introduced, without requiring any other components (Boyden et al. 2005). The term ‘optogenetics’ was coined in 2006 to describe the method of expressing genetically targeted photoreceptors in neurons, allowing for their selective activation or inhibition with light (Deisseroth et al. 2006). Subsequently, the achievement of mammalian behavioral control using optogenetic approaches rendered the microbial opsin approach promising (Aravanis et al. 2007). Soon after, the development of a major optogenetic tool, ‘Opsins’, occurred rapidly. This included the use of opsins such as archaea-halorhodopsin (NpHR, a hyperpolarizing chloride pump) and archaerhodopsin (a hyperpolarizing proton pump) (Zhang et al. 2007; Chow et al. 2010). Furthermore, channelrhodopsins have been altered to operate as bistable channels, and channelrhodopsin-2 (ChR2) has been engineered to act as an inhibitory channel (Berndt et al. 2009, 2014).

The study of neural circuits has undoubtedly contributed to the understanding of the mechanisms of epilepsy, pushing the development of relevant drugs and the treatment of epilepsy. A detailed summary of these techniques is presented as follows.

## Techniques Applied in the Study of Neural Circuits in Epilepsy

**Fig. 2:**
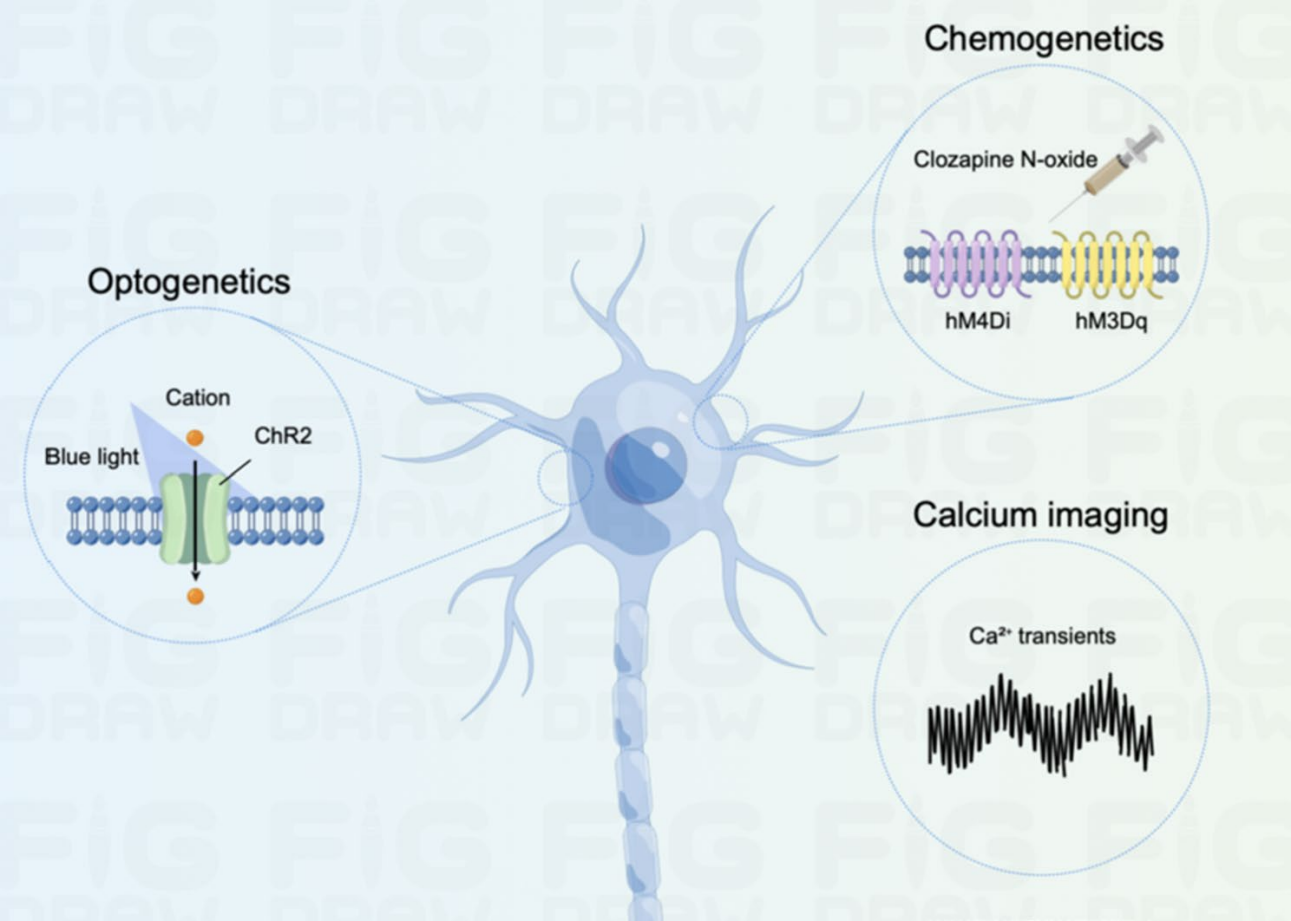
Several common techniques and their mechanisms. Optogenetics and chemogenetics are amenable for the manipulation of neural circuits through light-activated channels, such as channelrhodopsin-2, and via chemically activated designer receptors exclusively activated by DREADDs, like hM4Di and hM3Dq. Functional activities in neurons can be detected by calcium imaging, showing changes in calcium signals (via genetically encoded calcium indicators)

In an effort to advance our understanding of epilepsy and devise more effective treatment modalities, researchers are focusing their efforts on understanding the neural circuits implicated in this disorder. Technological advancements and innovations in neuroscience have enabled the application of diverse investigative techniques, such as optogenetics, calcium imaging, and chemogenetics (Fig. 2), facilitating a comprehensive analysis of neural circuits involved in epilepsy. These methodologies provide invaluable insights into the structure and functionality of these circuits, expanding our understanding of epilepsy and paving the way for the development of more effective treatment.

## Electrophysiology

Electrophysiology can thoroughly analyze the functional connections within complex neural circuits composed of various cell types, serving as an invaluable tool for investigating and interacting with the electrical aspects of living cells, tissues, and organisms (Jamieson and Piet 2022). It has critical applications in neuroscience where it evaluates neuronal electrical activity, illuminating aberrant neural processes that occur in complex diseases, including epilepsy (Table 2). Current studies have prioritized the refinement of electrophysiological methods, including the alteration of firing patterns and the development of superior electrodes for optimized signal detection. For instance, Sorokin et al. examined the firing patterns of thalamocortical relay cells in atonic epilepsy, demonstrating that a rapid shift to tonic discharge effectively halted absence epilepsy (Sorokin et al. 2017). This research method offers valuable insights for subsequent studies on related neural networks. Technological innovations, including bio-nano-modified microelectrode arrays and flexible graphene depth neuroprobes, have augmented the stability, precision, and utility of electrophysiological techniques in epilepsy researches. Moreover, electrophysiological methods are increasingly used to investigate the effects of epilepsy treatment medications. Jiang et al., for instance, discovered that low doses of lithium mitigated seizure susceptibility and severity, whereas high doses intensified these effects (Jiang et al. 2018a). Evaluating brain activity patterns through electrophysiological screening and pinpointing drug targets can potentially facilitate the prediction of drug efficacy and adverse effects, thereby advancing drug development (Heuzeroth et al. 2019; Xu et al. 2019).

**Table 2:** Electrophysiology in the studies of neural circuits in epilepsy

Types of epilepsy	Animal models	Methods inducing epilepsy	Related neural circuits	References
Epilepsy	FoxG1 + / − mice		Spontaneous glutamatergic transmission	Testa et al. (2019)
Repeated seizure	Adult male Sprague–Dawley rats	Cell type-specific, optogenetic stimulations	Whole-brain mapping of circuit dynamics	Choy et al. (2022)
Epilepsy	Nedd4-2andi rats (Male mice at age 4-weeks old)	Kainic acid	Ubiquitination of GluA1 subunit of the AMPAR	Zhu et al. (2017)
Febrile status epilepticus	C57BL/6 J male mice	Kainic acid	Pro-epileptogenic network	Chen et al. (2021)
TLE	Male Sprague–Dawley CD rats (150–175 g)	Kainic acid (15-mg/kg)	Hippocampal circuit	Arnold et al. (2019)
DS	Juvenile mice	Hyperthermia	Hippocampal synapse and circuit dysfunctions	Gu et al. (2014)

In the realm of clinical electrophysiological researches on epilepsy, the focus lies on electrically active cell membranes and their related components. A recent study revealed that chemogenetic silencing of hippocampal CA2 pyramidal cells significantly mitigated spontaneous seizures, indicating that CA2 might play a role in temporal lobe seizures (Whitebirch et al. 2022). Korgaonkar et al. discovered that the immune receptor TLR4 directly influenced post-traumatic neuronal excitability, suggesting that TLR4-mediated signaling might be a viable target to inhibit epileptogenesis following a traumatic brain injury (Korgaonkar et al. 2020). Additionally, Jiang et al. demonstrated that activating cortical SOM-INs could avert motor seizures and that mTOR-dependent dendritic inhibition remodeling could affect the seizure phenotype in generalized epilepsy (Jiang et al. 2018b). This suggests that mTOR inhibitors may not be universally effective as antiepileptic treatment. Furthermore, researchers also combined fMRI and electrophysiology to unveil novel mechanisms of seizure maintenance and interhemispheric propagation (Choy et al. 2022).

Electrophysiology also faces a number of challenges, such as insufficient resolution and a relatively homogenous range of brain regions to monitor. In response, researchers are continuously improving their experiments to achieve precise studies of functional connectivity within and between complex neural circuits composed of different cell types (Chou et al. 2022). High-resolution and wide-area monitoring deserves continued in-depth research in the future. In summary, electrophysiology is critical in elucidating epileptic neural circuits. Its growing prominence underscores the necessity of ongoing enhancements in electrophysiological methodologies and technologies, which will undoubtedly enrich our understanding of epilepsy and aid in the development of more effective therapeutic strategies.

## Calcium Imaging

As a classic technique, calcium imaging is a tool favored by neuroscientists for its ability to monitor large in vivo neural populations across the periphery with single neuron and single spike resolution (Robbins et al. 2021; Vogt 2021). It employs calcium ion markers to probe calcium concentrations within tissues. Primarily applied in neuroscience, calcium imaging enables the monitoring of calcium ion changes across numerous neurons, thereby revealing neuronal activities (Serrat et al. 2022). This conversion of elusive neural activity into tangible, animated displays is frequently used in studying neural circuits associated with epilepsy. Table 3 summarizes the application of calcium imaging in epileptic neural circuits in recent years.

**Table 3:** Calcium imaging in the studies of neural circuits in epilepsy

Types of epilepsy	Animal models	Methods inducing epilepsy	Related neural circuits	References
TLE	Male adult wild-type Swiss mice and GAD67-Cre mice		Modified inhibitory circuitry in CA1 neural circuit	Muldoon et al. (2015)
Post-traumatic epilepsy	Model of post-traumatic epilepsy in mice	Organotypic hippocampal slice culture	Changes inducing pathology among large populations of neurons	Lau et al. (2022)
Epilepsy	Larval zebrafish model	STXBP1 mutations	Spontaneous hyper-synchronized neuronal ensembles	Liu et al. (2021)
Recurrent seizures	Scn1a ± mice	Targeted deletion of exon 1 of the *Scn1a* gene, PV-Cre mice	GABAergic interneurons	Tran et al. (2020)
Focal Epilepsies (Particularly TLE)	Adult mice (50 days, > 20 g)	Pilocarpine hydrochloride (335 mg/kg)	Ca^2+^-channel upregulation in hippocampal CA1 neurons	Kulbida et al. (2015)
MTLE	Mouse	4-AP	A transition among states in hippocampal CA1 neurons	Mulcahey et al. (2022)
Epilepsy	Larval zebrafish		Network dynamics and neuronal ensembles	Liu and Baraban (2019)
Epilepsy	Mouse model of GBM (3 months old)	Targeted knockout mutation of the MapT gene	Reproducible progression from hyperexcitability to convulsive seizures	Hatcher et al. (2020)
Epilepsy	Anesthetized and awake mice	4-AP (15 mM, 500 nl) or picrotoxin (10 mM, 500 nl)	GABAergic interneurons	Wenzel et al. (2017)
Epilepsy	Gabrg2 knockout zebrafish	Switching on the diode light for at least 1 min	GABAergic and glutamatergic circuits	Liao et al. (2019)

Several studies have indicated that seizures result from modifications in the neural network, rather than the malfunction of isolated neurons. Wenzel et al. employed swift in vivo two-photon calcium imaging in the cortices of male rats, positing a two-stage model for the progression of focal seizures: initiation of micro-seizures followed by grand mal seizures via traveling waves in the adjacent cortex (Wenzel et al. 2019). Such investigations have offered a broader perspective on the intricate patterns of seizure microprogression.

Dravet syndrome (DS), a neurodevelopmental disorder typically accompanied by intractable epilepsy, has been studied using bimolecular calcium imaging on DS mouse brain sections (Chilcott et al. 2022). Findings suggest that cortical hippocampal circuits in DS mice are crucial pathological sites, emphasizing a dominant inhibitory effect of parvalbumin inhibitory interneurons (PV-INs) (Mattis et al. 2022). The dysfunction in PV-IN synchronization may play a pivotal role in the transition to a seizure state during temperature-induced seizures in DS, offering potential therapeutic avenues for seizure management (Tran et al. 2020). Further research by Somarowthu et al. revealed differential recruitment of distinct subclasses of interneurons, shedding light on the seizure mechanisms implicated in DS (Somarowthu et al. 2021). Researchers employing calcium imaging, in combination with high-throughput behavioral tracking and extracellular local field potential recordings, among other techniques, have identified spontaneous hyper-synchronous neuronal clusters during seizures (Myren-Svelstad et al. 2022). Their findings attribute the swift decrease in neural activities and subsequent depressive states following light-induced responses to homeostatic mechanisms triggered by excessive neural activities. These studies are instrumental in designing personalized epilepsy treatment plans.

GABAergic neurons are garnering significant attention owing to their essential function in epileptogenesis. Specific γ_2_ subunit knockdown of the GABA-A receptor was found to induce transient light-induced reflex seizures in zebrafish larvae (Liao et al. 2019). Conversely, GABA-B activation can regulate diverse receptors on excitatory glutamatergic neurons through both pre- and postsynaptic GABA-B interactions. It can also stimulate excitatory intermediates by activating pre- and postsynaptic GABA-B receptors, relieving inhibition on principal cells, and consequently enhancing the activity of excitatory neurons (Gerrard et al. 2018). Muldoon et al., utilizing a combination of in vivo two-photon calcium imaging and multi-unit extracellular recordings, found that in a chronic epilepsy model, GABAergic neurons in the CA1 region of the mouse hippocampus were preferentially recruited during spontaneous interictal activity (Muldoon et al. 2015). They proposed that this abnormal, unbalanced recruitment might have significant downstream consequences with potential clinical implications.

However, calcium imaging has limitations in terms of its observational breadth and imaging depth (Ait Ouares et al. 2020). Currently, full-brain calcium imaging is only achievable in small organisms like zebrafish larvae, nematodes, and fruit flies, or is used for in vitro cell observation. It is not applicable to living organisms with larger brain tissues or obstructed by skulls (Wong 1998; Nietz et al. 2022). In conclusion, calcium imaging is instrumental in identifying the network nodes outside the seizure locus that are responsible for seizure initiation, propagation, and termination. It plays a significant role in the detection of calcium ions in organisms at cellular and even subcellular structure levels, providing high spatial and temporal resolution.

## In Vivo Microdialysis

In vivo microdialysis serves as an essential technique for sampling the immediate vicinity of the catheter tip to identify abnormal concentrations of specific molecules in the extracellular milieu of the brain (Zestos et al. 2019). These abnormal concentrations can provide insights into the etiology of numerous brain diseases, including Huntington's disease, Parkinson's disease, Alzheimer's disease, schizophrenia, traumatic brain injury, stroke, depression, and epilepsy (Di Giovanni et al. 2009; Zestos et al. 2019; Todd and Butterworth 2001; Bjorkli et al. 2021; McAdoo and Wu 2008) (Table 4).

**Table 4:** In vivo microdialysis in the studies of neural circuits in epilepsy

Types of epilepsy	Animal models	Methods inducing epilepsy	Related neural circuits	References
MTLE	Male Sprague–Dawley rats	Methionine sulfoximine (2.5 mg/mL)	GABAergic neurotransmission	Dhaher et al. (2021)
Autosomal dominant sleep-related hypermotor epilepsy	S286L-TG rats		Transmission abnormalities of L-glutamate and GABA in the thalamocortical pathway	Fukuyama et al. (2020b)
Autosomal dominant sleep-related hypermotor epilepsy	S286L-TG rats		Transmission in the RTN–MDTN–OFC pathway associated with connexin hemichannel	Fukuyama et al. (2020a)
Epilepsy	Thy1-ChR2 rats	Optogenetic stimulation	Acquired hyperexcitable circuit	Shimoda et al. (2022)

In vivo microdialysis has emerged as an essential tool in revealing key aspects of epilepsy, particularly in showing how treatment can influence seizure activity (Zestos et al. 2019). This technique has been instrumental in tracing changes in endogenous substances, such as excitatory and inhibitory neurotransmitters and their metabolites, at various stages: before, during, and after seizures. Luna-Munguia et al. integrated state-of-the-art microdialysis techniques with an innovative model of ictogenesis, enabling simultaneous monitoring of 24 molecules during ictogenesis to discern the chemical shifts associated with epileptogenesis and ictogenesis (Luna-Munguia et al. 2019). Similarly, Fukuyama et al. utilized multiprobe microdialysis in conjunction with ultra-high-performance liquid chromatography to examine transmission abnormalities of L-glutamate and GABA in the thalamocortical pathway of transgenic rats bearing rat S286L-mutant Chrna4 (S286L-TG) (Fukuyama et al. 2020b). In a separate study, the same team implanted a concentric direct-insertion type dialysis probe in the orbitofrontal cortex of rats after post-AMPA-evoked stimulation (Fukuyama et al. 2020a). Dhaher et al. merged brain microdialysis with mass spectrometry to investigate seizure initiation, seizure propagation, and extracellular brain levels of glutamate and GABA (Dhaher et al. 2021). Besides, Shimoda et al. employed a microdialysis probe inserted into the CA3 hippocampus via a guide cannula, preserving collected samples using liquid chromatography-tandem mass spectrometry (Shimoda et al. 2022).

The main limitations of in vivo microdialysis are the size of the probe and the damage to the tissue caused by inserting the probe. Besides, since certain neurotransmitters are low in brain dialysate, samples must be taken at regular intervals, a time scale which is far removed from the time scale of neuronal events (Holmgaard et al. 2010; Zestos et al. 2019). For in vivo neurochemical monitoring, microdialysis is often paired with chromatographic, electrophoretic, or enzymatic assays (Tan et al. 2021). Of these, liquid chromatography, mass spectrometry, and their tandem applications are most frequently employed for a detailed examination of in vivo neurochemistry during seizures (Kennedy 2013). Given the challenges related to optogenetic neurochemical monitoring, such as the requirement for high sampling rate analytical methods and in vivo neurochemical monitoring during optogenetic neuronal stimulation, the fusion of optogenetics with microdialysis sampling might offer an enlightening approach to measure neurochemical alterations before, during, and after controlled epileptic seizures (Parrot et al. 2015).

## Fiber Photometry

In recent years, fiber photometry has become a popular technique for in vivo neural recording, favored for its simplicity and specificity (Legaria et al. 2022). Its primary aim is to elucidate the real-time functions of distinct neural pathways in mammalian behavior by optically recording natural neural activity in genetically specified and connectivity-defined projections (Zhang et al. 2021a). The sensitivity of this recording technique enables the in vivo direct measurement of coordinated neuronal afferent activities projecting to a specific downstream target deep within the brain of a mammal engaged in behavior. This is achieved by the application of genetically encoded Ca^2+^ indicators and advanced photometric devices (Legaria et al. 2022). Table 5 summarizes the applications of Fiber photometry techniques in epilepsy neural circuits in recent years.

**Table 5:** Fiber photometry in the studies of neural circuits in epilepsy

Types of epilepsy	Animal models	Methods inducing epilepsy	Related neural circuits	References
TLE	C57BL/6 mice	KA (15 mg/kg)	CA1, CA3, and DG of the hippocampus, as well as the entorhinal cortex	Zhang et al. (2019b)
TLE	C57BL/6 J mice	Stimulated the CA3 of mice with a constant‐current stimulator	DR 5-HTergic system	Cui et al. (2014)
TLE	CR-cre mice and C57BL/6 J mice	Stimulated the CA3 of mice with a constant‐current stimulator	Downstream neural circuits of PIL CR neurons	Qi et al. (2022)
TLE	CR-cre mice and C57BL/6 J mice	Stimulated the CA3 of mice with a constant‐current stimulator	PIL-lateral amygdala CR circuit; PIL-zona incerta CR circuit	Qi et al. (2022)
TLE	kainate model of adult mice (Mus musculus)	KA (200 nL of 2.5 mM into the right CA1)	Hippocampal circuitry including CA3 tracts extending to the dorsoventral portion of the brain	Murphy et al. (2022)

Utilizing the power of fiber photometry-based calcium recording, Zhang et al. analyzed the relationship between epileptic seizures and brain calcium activities in freely behaving mice, presenting a simple procedure with minimal damage to brain tissues (Zhang et al. 2019b). Additionally, Cheng et al. integrated optogenetics and DBS with fiber photometry, using a fiber photometry system (Nanjing Thinkertech), during hippocampal kindling (Chen et al. 2020). They examined the role of dorsal raphe and its 5-HTergic neurons in epilepsy by bandpass filtering and collecting GCaMP fluorescence with a photomultiplier tube. In a separate study, researchers combined fiber photometry with c-fos mapping to identify numerous hyperactivated CR neurons in the PIL. This approach helped to investigate the causal roles of these neurons in hippocampal seizures (Qi et al. 2022). To further illustrate the utility of fiber photometry, Xu et al. combined in vitro c-fos staining with in vivo fiber photometry recordings to monitor activity changes in the paraventricular thalamic nucleus (Xu et al. 2022). Besides, Murphy et al. pioneered a new tool termed the Fiber Photometry Coupled Focused Ultrasound System (PhoCUS), which allows for simultaneous monitoring of focused ultrasound effects on neural activities of subcortical genetically targeted cell types in freely behaving animals (Murphy et al. 2022). This advancement provides a new way to investigate focused ultrasound effects on specific cell types in deep brain regions.

Evidently, fiber photometry offers a promising method for advancing studies into the pathogenesis of epilepsy and epileptiform signal transmission. This is attributable to its exceptional sensitivity and high-resolution detection capabilities, especially regarding calcium signal detection in multiple brain regions. However, similar to electrophysiology, fiber photometry faces the problems of low resolution and limited range of brain area monitoring, and its difficulty in controlling the depth of captured signals, which are worthy of subsequent research and improvement (Mineur and Picciotto 2023).

## Virus Tracing Technology

Due to the collaborative efforts across various disciplines, particularly the combined progress in molecular biology, virology, microscopy, computer science, and genetics, effective advancements have been achieved in the methodological aspects of neuron tracing. Viral tracing techniques are a hallmark product of this progress. In the past twenty years, viral tracing techniques have garnered a surge of interest in neural circuitry studies, especially in epilepsy studies (Table 6). They exhibit a unique ability to deliver tracers in a cell-type-specific and circuit-selective fashion, employing cis-, retrograde, and trans-synaptic methods (Liu et al. 2022).

**Table 6:** Virus tracing technology in the studies of neural circuits in epilepsy

Types of epilepsy	Animal models	Methods inducing epilepsy	Related neural circuits	References
mTLE	mTLE rats	Pilocarpine hydrochloride (340 mg/kg)	Substantial remodeling hippocampal circuit	Du et al. (2017)
Chronic TLE	Female Kunming mice (Four-week-old, 25–30 g)	Pilocarpine (300 mg/kg)	Neurogenesis in the DG	Hu et al. (2015)
Spontaneous recurrent seizure	Genetically engineered mice (hM4Difl/ +)	Pilocarpine (290 mg/kg)	DGC-related neural circuits	Zhou et al. (2019)
Focal epilepsy and drug-resistant epilepsy	Adult male Sprague–Dawley rats (210–230 g, 8 weeks)		Hippocampal neural circuits	Yang et al. (2022)
Epilepsy	Cyfip2 + / − mice		Prefrontal cortex neural circuits	Lee et al. (2020)
Epilepsy	Retrovirus infection models		Rhythmic neural circuits	Sivaramakrishnan and Lynch (2017)
TLE	Male Vgat-Cre mice (2–4 months)	Ten kindling stimulations daily	Long-range SNr-PF disinhibitory circuits	Chen et al. (2020)
TLE	Male Swiss mice (25–30 g)	Pilocarpine or saline (300 mg/kg)	Dentate gyrus neural circuits	Chen et al. (2023b)

Innovative studies have applied rabies tracing techniques to explore neural circuits relevant to epilepsy with some success. Studer et al. utilized rabies virus-mediated retrograde synaptic tracing to investigate a mouse model of atonic epilepsy (Studer et al. 2022). Their results showed that cortical neurons, located in both the superficial and deep layers of the whisker primary somatosensory cortex, demonstrated significantly increased and abnormal connectivity in a genetic model of anhedonic epilepsy. This suggests that the enhanced structural connectivity patterns of these neurons might be a crucial pathological basis for increased neuronal synchronization and the generation of anhedonic seizures. In sum, this research offers new insights into the relationship between neural hypersynchronization and abnormal structural connectivity in neuronal networks during epileptic seizures.

Rabies virus-mediated presumptive retrograde trans-synaptic tracing is frequently combined with other tracing techniques for improved performance, such as alternative viral tracing methods, optogenetics, and chemogenetics (Islam et al. 2022; Kato and Kobayashi 2020; Wang et al. 2020). In an experimental rat model of medial temporal lobe epilepsy (mTLE) induced by trichothecene, Du et al. implemented a dual viral tracing strategy, which combined retroviral birth dating with rabies virus-mediated presumptive retrograde trans-synaptic tracing (Du et al. 2017). They observed substantial remodeling of the hippocampal circuit following epileptogenic injury, causing DGCs to generate significant excitatory monosynaptic inputs, such as local recurrence and widespread feedback loops. A similar outcome was achieved by Zhou et al., who integrated rabies virus tracing techniques with chemogenetic approaches (Zhou et al. 2019). Future research could consider utilizing other tracing techniques or examining unexplored subtypes of interneurons.

Recently, Wang et al. integrated optogenetics and chemogenetics with a combination of in vivo and in vitro electrophysiology, as well as retrograde rabies virus tracing (Wang et al. 2020). This innovative combination helped to elucidate a direct septo-hippocampal cholinergic circuit, which efficiently mitigates seizures by instigating growth inhibitor inhibition in an animal model of TLE. This study improves our understandings of alterations in seizure circuits and their precise spatiotemporal controls. Despite these advancements, few studies have documented brain circuits activities during seizures through recombinant adenoviral methodologies. Currently, lentiviruses and rabies viruses are widely used in the tracing of neural circuits (Kato and Kobayashi 2020; Deng et al. 2022). It is crucial to choose different viral vectors based on specific research objectives.

There are also several limitations in virus tracing technologies. Due to the specificity of the vector, it often faces biosafety issues such as neurotoxicity (Sun et al. 2019b). In addition, the relatively high cost and strict laboratory requirements also limit the widespread application of virus tracing technology to a certain extent (Li et al. 2020). Despite these disadvantages, overall, virus tracing technology remains a widely applicable technique at present.

## Optogenetics

Optogenetics combines optical and genetic techniques by genetically introducing suitable exogenous light-sensitive proteins into specific active cells (Duebel et al. 2015). By stimulating these light-sensitive proteins with light of a specific wavelength, through genetic methods, it modulates the activity of neurons and controls the switch for cellular and animal behavior. Optogenetics is an innovative technology which integrates principles from optics, software control, genetic engineering, and electrophysiology to simulate neural activities concerning timing, magnitude, and individual cellular patterns (Hoffman et al. 2022). This methodology facilitates an invasive investigation of the links between distinct neural circuits and brain functions (Table 7). Since the seminal publication by Deisseroth, optogenetics has evolved into an essential tool in neuroscience research (Deisseroth 2011; Fenno et al. 2011). It has proved invaluable in uncovering intra- and extracellular activities within the neural circuits associated with epilepsy.

**Table 7:** Optogenetics in the studies of neural circuits in epilepsy

Types of epilepsy	Animal models	Methods inducing epilepsy	Related neural circuits	References
TLE	Anesthetized mice	Kindling and kainic acid	SNr and PF disinhibitory circuit involving SNr + PV GABAergic neurons and posterior PF GABAergic neurons	Chen et al. (2020)
Absence epilepsy	Male GAERS rats		Long-range subcortical regulation of serotonergic neuromodulation	Sere et al. (2021)
Status epilepticus	Male mice	Pilocarpine	Mossy cell circuit	Butler et al. (2022)
TLE	DrD2-Cre transgenic mice	Pilocarpine	Glutamatergic mossy cell circuit	Botterill et al. (2019)
Epilepsy	Mice		Epileptic cortical circuits	Yang et al. (2021)
TLE	TLE mouse		Direct septum-hippocampus cholinergic circuit	Wang et al. (2020)
Epileptic seizures	Zebrafish (4–5-weeks-old)		Glia-glia and glia-neuron connections	Diaz Verdugo et al. (2019)
mTLE	adult transgenic male mice (8–12 weeks)	KA or saline	Hippocampal sclerosis	Paschen et al. (2020)
TLE	Male and female VGAT-IRES-Cre mice (6 to 18-week-old)	Kainate (100 nl, 20 mM in saline)	Medial septal GABAergic neurons-modulated oscillations across multiple hippocampal locations	Hristova et al. (2021)
Epilepsy	CR-cre mice	Kindling	CR circuit	Qi et al. (2022)
Absence Epilepsy	Tottering Mice		Cerebellar nuclei neurons	Eelkman Rooda et al. (2021)

Optogenetic tools have enabled an unprecedented degree of functional circuits mapping in epilepsy research. Temporal lobe epilepsy (TLE), one of the most common forms of focal epilepsy, has been extensively probed by optogenetics (Mokhothu and Tanaka 2021; Christenson Wick et al. 2017). Chen et al. investigated the complex circuitry of TLE, suggesting that the long-range SNr-PF inhibitory circuit is involved in regulating seizures in TLE and the deactivation of this circuit could lessen seizure severity (Chen et al. 2020). In a comparative study of pathological changes in the medial septum's (MS) hippocampal circuits between TLE patients and healthy individuals, Wang et al. discovered that direct MS-hippocampal cholinergic projections mediated antiepileptic effects by preferentially targeting hippocampal GABAergic neurons, underscoring the specific role of the MS-hippocampal cholinergic circuit in TLE (Wang et al. 2020). Additionally, aberrant sparse activity of hippocampal dentate gyrus (DG) granule cells (GCs) has been found to contribute to TLE, with recent optogenetic evidence indicating that the axons of glutamatergic mossy cells play a pivotal role in this process (Botterill et al. 2019). These studies hold significant potential for precisely targeting epilepsy.

Furthering the integration of optogenetics with viral tracing techniques, Sere et al. investigated the role of lateral hypothalamic neurons (LH) in dyscalculia epilepsy (Sere et al. 2021). The study illustrated that LH neurons extend to the dorsal raphe nucleus (DRN) in the brainstem, with a subset being GABAergic. It confirmed the presence of long-distance subcortical modulation of serotonergic neuromodulation during anhedonic seizures, enhancing our understanding of seizure complexities. It is known that pulmonary portal mossy cells can influence hippocampal circuits function, and substantial loss of these cells is associated with alterations in the same circuits during epileptic states. Optogenetics has been instrumental in delineating this involved microcircuitry (Butler et al. 2022). Seeking to clarify how abnormal neural circuits contribute to epilepsy, Yang et al. examined circuit mechanisms in a mouse model of focal cortical malformations through optogenetic stimulation (Yang et al. 2021). Their findings revealed site-specific and cell-type-specific synaptic reorganization within epileptic cortical circuits, highlighting the need for more extensive research to understand the prevalence and overall landscape of these abnormal circuits. Besides, recent work has demonstrated that calretinin (CR) neurons in the posterior intralaminar thalamic nucleus (PIL) exhibit varying influences on hippocampal seizures via distinct downstream circuits, adding to our understanding of the PIL-lateral amygdala CR circuit (Qi et al. 2022).

Optogenetics offers the ability to accurately activate or inhibit-specific neurons, serving as a research tool and potential therapeutic strategy for managing aberrant neuronal activities. GABAergic neurons have been proposed as novel targets for optogenetic modulation of temporal lobe seizures. As the scope of animal studies and clinical treatment broadens, optogenetics holds considerable promise in elucidating epileptogenesis, seizure onset, and pathological circuits, which could potentially open avenues for alternative therapeutic strategies such as gene therapy. The study of neuronal interactions during seizures with the aid of optogenetics has garnered increasing interest and is expected to become a central area of investigation in epilepsy research.

Optogenetics also faces some dilemmas. The expression of photosensitive proteins is not uniform across neuronal populations, which can lead to heterogeneity in the magnitude and scope of optogenetic manipulation (Melonakos et al. 2020). In addition, the simultaneous action of a large range of light stimuli on a population of neurons may cause circuits to show non-physiological patterns of activity (Peralvárez-Marín and Garriga 2016). And because of the complexity of neural circuits, optogenetics is more difficult to apply to the primate brain, which limits its possible wide application (Merlin and Vidyasagar 2023).

## Chemogenetics

Similar to optogenetics, chemogenetics is widely applied in neuroscience for the modulation of specific neurons or neural circuits. Chemogenetics is a method which utilizes gene technology and artificially designed drugs to alter the function of neurons (Poth et al. 2021; Magnus et al. 2019). It is commonly employed to explore the function and activity dependence of neurons. This technique involves the introduction of engineered receptors or channels, which selectively respond to synthetic ligands, thereby modulating cellular signaling pathways. Table 8 summarizes the recent applications of chemogenetics in the neural circuits of epilepsy. Chemogenetics can either be direct, through the utilization of synthetic ligand-gated ion channels, or indirect, by leveraging DREADDs, tools derived from G-protein-coupled receptors (Oyrer et al. 2018). DREADDs have gained popularity as tools of chemogenetics in studying the neural circuits of epilepsy due to their capability to either inhibit or excite neurons. This is achieved through the expression of designer receptors which respond to exogenous compounds such as clozapine N-oxide or olanzapine.

**Table 8:** Chemogenetics in the studies of neural circuits in epilepsy

Types of epilepsy	Animal models	Methods inducing epilepsy	Related neural circuits	References
TLE	Thy1-ChR2 transgenic mice	Optogenetic train stimulation	Local and transhemispheric circuits	Berglind et al. (2018)
Chronic, drug-resistant epilepsy and acutely triggered seizure activity	organotypic hippocampal slices	clozapine N-oxide (4 mg/kg)	Parvalbumin, SST, and vasoactive intestinal peptide expressing interneurons	Cǎlin et al. (2018)
TLE	Intrahippocampal mouse model	Clozapine-N-oxide (1, 3, and 10 mg/kg) and clozapine (0.03 and 0.1 mg/kg)	Excitatory pyramidal and granule cell neurons of the sclerotic hippocampus	Desloovere et al. (2019)
TLE	Wild-type C57BL/6 mice	Scopolamine (1 mg/kg) and pilocarpine (315 mg/kg)	The DG’s aggregate output	Kahn et al. (2019)
Alcohol withdrawal -associated seizures	C57BL/6 mice		Hippocampal neural circuits	Lee et al. (2019)
GABRG2‐deficient epilepsy	a Gabrg2 + /Q390X knock-in mouse and a Gabrg2 + / − knock-out mouse		GABAergic neurotransmission in central nucleus of the amygdala	Zhang et al. (2019a)
Spontaneous recurrent seizures	C57BL/6 mice	pilocarpine (290 mg/kg)	Newborn hippocampal DGCs in pro-epileptic neural circuits	Zhou et al. (2019)
TLE	C57BL/6 J mice	Hippocampal kindling model by a constant current stimulator; intra-hippocampal KA model (0.5 μg/μL, 0.6 μL)	A nigra-parafascicular disinhibitory circuit for regulation of seizure in TLE	Chen et al. (2020)
TLE	Mice	Kindling- and KA-induced epileptic models	MS-hippocampus cholinergic circuit in TLE	Wang et al. (2020)
TLE	Male Sprague–Dawley rats	Intraperitoneal KA rat model	Excitatory hippocampal neurons	Goossens et al. (2021)
Childhood absence epilepsy	C57BL/6 mice	Pentylenetetrazol (3, 2, and 1 mg/ml)	Cortico-thalamocortical network	Panthi and Leitch (2021)
SCN8A epileptic encephalopathy	patient-derived SCN8A mutated mouse model of SCN8A epileptic encephalopathy		Various cortical and subcortical neural circuits	Wengert et al. (2021)
TLE	C57/BL6J mice	KA (0.3 mg/mL in 0.1 M)	dHPC dorsal lateral septum circuit	Cao et al. (2022)
TLE	Male C57BL/6 Hsd mice	Intrahippocampal kainic acid injection	Epileptic network	Desloovere et al. (2022)
Focal cortical epilepsies	Gad2-IRES-Cre mice targeted hM4D expression to either the medial prefrontal cortex or the barrel cortex		The barrel cortex and the medial prefrontal cortex	Goldenberg et al. (2023)
TLE	C57BL/6 J mice	Intra-amygdala KA model (0.2 µg in 0.2 µl ACSF) and lithium chloride –pilocarpine model (10 mEq/kg)	D1R-MSN and D2R- MSN	Zou et al. (2022)
TLE	Rats	Kindling model	Projections from mediodorsal nucleus to prelimbic cortex	Wicker et al. (2022)

Chemogenetic tools have proven instrumental in the selective activation or silencing of pathways in a specific population of neurons. It has greatly facilitated the investigation of anatomically constrained neural circuits in epilepsy and the development of high-resolution circuit maps associated with seizures. Chemogenetics has illuminated the circuit-based mechanisms underlying seizures. For instance, Kahn et al. used DREADDs to manipulate the activity of dentate gyrus cells (DGCs) to target the aggregate output of the dentate gyrus, thereby demonstrating the importance of fine-tuning activity within this region for cognitive performance (Kahn et al. 2019). In addition, Zhou et al. have underscored the crucial role of newborn DGCs in the hippocampus in the formation of epileptic neural circuits and the initiation of spontaneous recurrent seizures (Zhou et al. 2019). Through the application of chemogenetic methodologies, circuits such as the nigra-parafascicular disinhibitory circuit, which regulates seizures in TLE, and the dHPC dorsal lateral septum circuit that impairs risk assessment behavior, have been identified and evaluated (Chen et al. 2020; Cao et al. 2022). Projections from the mediodorsal nucleus to the prelimbic cortex have also been implicated in the propagation of amygdala-kindled seizures (Wicker et al. 2022). An increasing number of studies are leveraging chemogenetics for the specific activation and inhibition of neurons associated with particular circuits, thus assessing the impact of chemogenetic interventions on epileptic activity (Berglind et al. 2018). Furthermore, developing novel DREADDs ligands and investigating new mouse models are subjects of current research, and improvements in these areas are anticipated (Desloovere et al. 2022).

Interneurons have garnered attention as pivotal components for understanding the neural circuits involved in epilepsy, with particular focus on the three main interneuron populations in the rodent hippocampus: parvalbumin (PV), somatostatin (SST), and vasoactive intestinal peptide expressing interneurons (Miles et al. 1996; Paz and Huguenard 2015; Lovett-Barron et al. 2012). Cǎlin et al. utilized excitatory DREADDs to activate different interneuron subtypes, finding that their ability to increase postsynaptic inhibition in principal neurons and reduce epileptiform synchronization in neuronal networks varies. However, all these subtypes may serve as effective anticonvulsant strategies (Călin et al. 2018). Besides, Panthi and Leitch focused on the activation of PV + interneurons using DREADDs, and they administered clozapine N-oxide to activate these interneurons (Panthi and Leitch 2021). Their study suggested that FFI of PV + interneurons within cortico-thalamocortical microcircuits could inspire new advancements in anti-absence seizure treatment. A separate study emphasized the role of DG PV-INs, proposing that the inhibitory function of PV-INs could be a promising therapeutic target for seizure modulation (Mattis et al. 2022). Notably, GqDREADD-mediated chemogenetic activation of wild-type SST interneurons could lead to an increase in spontaneous excitability and susceptibility to depolarization block, thereby contributing directly to seizures in vivo and highlighting the substantial role of SST interneurons in seizure events (Wengert et al. 2021).

Moreover, the field of chemogenetics is proving to be groundbreaking in making strides toward clinical translation in the treatment of epilepsy. Gene therapy, which enables the manipulation of neuronal activities in the epileptic focus while simultaneously preserving function, appears as a promising alternative to traditional pharmacological methods and surgical interventions for epilepsy. By expressing proteins that can be regulated by drugs, chemogenetics can serve as a titratable therapy that could potentially overcome challenges associated with traditional therapies that are limited by fixed gene dose, expression of that gene, and the distribution of transfected cells (Kullmann et al. 2014; Walker and Kullmann 2020). Zhang and Wang have summarized and elucidated the benefits of chemogenetics for gene therapy and several studies have demonstrated the potential effectiveness of chemogenetics in epilepsy treatment, thereby supporting its prospective clinical translation in the future (Zhang and Wang 2021).

Chemogenetics could result in minimal damages to animals. After viral expression in the brain, each experiment only requires the injection of drugs into muscles or veins to inhibit or excite a specific brain region, making it highly suitable for translational medical research (Song et al. 2022). However, compared to optogenetics, chemogenetics also has its drawbacks, particularly in terms of lower temporal precision. The effects of drugs persist for several hours, during which neurons remain in an abnormal state. Cognitive processes in the brain often change rapidly, with multiple cognitive events occurring within seconds or even one second. Chemogenetics may struggle to separate these transient and sequentially occurring cognitive processes (Raper and Galvan 2022).

## In Vitro Models

In response to the growing sophistication of tools for the exploration of neural circuits in epilepsy, there is an emergent need for more advanced in vitro models. These models could serve to address current limitations and bolster biomedical research, with a particular focus on the discovery of antiepileptic drugs (Quadrato et al. 2017; Elder et al. 2022). Traditional murine models have demonstrated efficacy in elucidating the impact of epilepsy-associated genes on neural cells and circuits. However, they are unsuitable for high-throughput screening procedures and exhibit dissimilarities in brain development compared to their human counterparts (Silbereis et al. 2016; Jiang et al. 2018b). In efforts to overcome these challenges, human stem cell-derived models have been introduced. These models include neurons derived from iPSCs and organoids cultured from patient cells, both of which offer a patient-specific genetic background and human-specific cell types.

### iPSC-Derived Neurons

Over recent years, iPSCs have taken a prominent role in epilepsy research, given their inherent pluripotency which lends itself well to modeling neurological disorders such as epilepsy (Shi et al. 2012). It has been found that iPSCs could reproduce the genetic mutations and background of the affected brain cells, thus making them suitable for modeling these disorders (Bhargava et al. 2022). Following the initial success in generating iPSCs from mouse fibroblasts, significant advancements have facilitated their application in epilepsy pathogenesis studies (Karagiannis et al. 2019).

A variety of genetic mutations, especially in different ion channels, play a role in the development of epilepsy. These mutations can affect the excitatory/inhibitory (E/I) balance and the stability of neural circuits. Key channels involved include voltage-gated sodium, potassium, and calcium channels, along with ligand-gated glutamatergic and GABAergic receptors (Oyrer et al. 2018). However, few of these mutations have been examined in humanized in vitro models. With the application of epilepsy patient-derived iPSCs to generate both excitatory and inhibitory neurons, voltage-gated sodium channels (Nav), including Nav1.1, Nav1.2, and Nav1.6 (encoded by SCN1A, SCN2A, and SCN8A, respectively), which have been associated with epilepsy, have been examined for their essential role in the depolarizing phase of the action potential (Child and Benarroch 2014). While most studies are focusing primarily on SCN1A and only investigating Nav1.1 in vitro, Tidball et al. have developed iPSC lines from epilepsy patients with SCN2A and SCN8A LOF mutations for prospective studies (Kim et al. 2018; Tidball et al. 2017). Mutations in voltage-gated potassium channels, such as Kv7.2 and KNa1.1, have also been modeled in vitro (Simkin and Kiskinis 2018).

It is understood that the hippocampus' homeostatic set point is regulated by dihydroorotate dehydrogenase. However, maladaptive changes can readily disrupt this homeostatic plasticity. Mutations in ion channels and components of the mTOR pathway have surfaced as a primary area of focus for neural circuits research in epilepsy (LaSarge et al. 2021; Weng et al. 2022). The dynamic interactions between these components warrant further investigation through humanized in vitro systems. These systems offer a promising platform to identify homeostatic alterations within neural circuits, which could contribute to epileptogenesis.

There are also several limitations of iPSCs technology. For complex, multigenic epilepsy types, iPSCs technology is difficult to model effectively. In addition, the overall low reprogramming efficiency and the high cost used to produce and characterize each cell line discourage their application in experiments (Huangfu et al. 2008; Sahlgren Bendtsen and Hall 2023). More basic research is still needed to develop this technology.

### Organoids

2D cultures of iPSC-derived neurons can offer a valuable understanding of molecular mechanisms at the individual neuron level (Heider et al. 2021). However, they fall short in enabling investigation into neurogenesis and neuronal circuits. Moreover, they lack the 3D architecture characteristic of the brain and exhibit immature electrophysiological properties (Malik and Rao 2013). Organoid models effectively address these constraints, particularly in diseases associated with dysfunctional neural circuits, such as epilepsy (Andrews and Kriegstein 2022). Advances in human iPSC techniques have facilitated the development of 3D brain models, including brain organoids and cerebral organoids (Swingler et al. 2023). These models have often been employed for in vitro neuro-developmental research, but their potential in the context of epilepsy, especially for studying neural circuits, has been relatively underexplored (Pranty et al. 2022).

Birey et al. employed brain cortical spheroids (a specific type of cerebral organoid) to examine the developmental features of neurodevelopmental disorders, including epilepsy (Birey et al. 2017). Another study conducted by Blair et al. created cortical organoids from genetically engineered stem cell lines to investigate tuberous sclerosis (TS) complex (Blair et al. 2018). Additionally, Litwa further discussed the ability of brain organoids to reveal TS pathology, unravel disease mechanisms, and illuminate potential contributions of neurodevelopmental alterations to subsequent age-related neurodegeneration (Litwa 2022). Besides, Sun et al. developed 3D cortical organoids, which followed a normal developmental trajectory, to investigate whether functional deficits are relevant to the progression of Angelman syndrome and model epilepsy susceptibility in this syndrome (Sun et al. 2019a). Variations in neuronal network dynamics in numerous cells were monitored in both wild-type and knockout cortical organoids. In a study by Samarasinghe et al., calcium sensor imaging and extracellular recording techniques were used to analyze the functions of brain organoids at the network-level (Samarasinghe et al. 2021). Their research indicated that the fusion organoid system is amenable to detailed cellular and circuit analyses, providing insights into modeling human neurological disorders. Given the absence of in vivo connectivity, which hinders the integration with other circuits controlling behaviors, Revah et al. transplanted human stem cell-derived cortical organoids into the somatosensory cortex of newborn athymic rats (Revah et al. 2022). Their results demonstrated that these organoids were able to develop mature cell types and integrate into sensory and motivation-related circuits.

Although organoids have developed rapidly in recent years, for brain organoids, their development has not been smooth due to the lack of an in vivo brain tissue microenvironment, neuronal circuits, blood vessels, and immune system (Cakir et al. 2019; Dijkstra et al. 2018; Tao et al. 2022). How to systematize, vascularize, and immunize brain organoids is a focus of future research and holds infinite possibilities.

## Additional Considerations

As clearing methodologies continue to progress, the necessity for superior imaging techniques intensifies, with particular emphasis on speed and resolution. Light-sheet microscopy represents a promising technique that provides rapid imaging paired with high spatiotemporal resolution (Hillman et al. 2019). It differs from traditional confocal and two-photon microscopy as light-sheet microscopy employs optical sectioning coupled with illumination and fluorescence detection (Wan et al. 2019). It effectively renders detailed images of sizable, cleared, and expanded samples, making it particularly suitable for neuroscience applications such as in vivo imaging of mammalian brains (Fiolka 2021).

A robust light-sheet methodology is necessary to encourage further fundamental mechanistic studies of seizures and epilepsy. The selective plane illumination microscopy system, introduced by Huisken et al., has been extensively employed to image small, living organisms, such as *Drosophila* and zebrafish embryos (Huisken et al. 2004). In another study, a custom-built light-sheet microscope was applied to construct a brain atlas of larvae for assessing in vivo response of the vertebrate brain to drug treatment (Wolf et al. 2015). Besides, Rosch et al. developed their light-sheet microscope based on two-photon light-sheet imaging (Rosch et al. 2018). With an emphasis on elucidating aberrations across spatial scales, they imaged the larval zebrafish brain during acute epileptic seizures induced with pentylenetetrazole. Their results revealed pathophysiological causes of epilepsy, discovering that distinct shifts in local (intrinsic) and long-range (extrinsic) synaptic transmission dynamics may provide key insights into seizure pathomechanisms.

Tissue optical clearing aims to optically clarify samples, thereby providing three-dimensional data with high spatial resolution through deep fluorescent microscopic imaging (Liang and Luo 2021). Given the tissue damage caused by serial slice sectioning and subsequent reconstruction procedures and the limited imaging depths offered by techniques such as light-sheet, two-photon, and multiphoton microscopy, optical clearing provides practical solutions to these challenges. By addressing tissue heterogeneity and decreasing light scattering, which primarily contributes to the opacity of mammalian tissues, optical clearing procedures hold great potential to enhance light penetration depth in tissues, thereby increasing transparency. These technologies have all advanced our understanding of the neural circuits behind epilepsy.

## Integration of Techniques

In order to deepen the understanding of the neural networks implicated in epilepsy, a combination of techniques has been adapted in certain cases. The combination of these diverse approaches fosters a more holistic comprehension of the inherent mechanisms, providing potential insights for developing novel treatment aimed at managing this debilitating disorder.

Wang et al. employed a systematic and reasoned strategy, integrating a series of advanced methodologies such as MRI, DTI, optogenetics, chemogenetics, electrophysiology, and retrograde rabies virus tracing (Wang et al. 2020). This collection of techniques facilitated a thorough understanding of the MS-hippocampus cholinergic circuit's role in TLE. The incorporation of imaging, neuronal manipulation, and circuit tracing allows researchers to examine pathological alterations, manipulate-specific neurons, and trace circuit connections, ultimately resulting in solid conclusions regarding the circuit's functionality and its prospective therapeutic significance in epilepsy. In a similar vein, Sere et al. investigated the role of the LH in absence epilepsy through the utilization of a blend of viral tract tracing, optogenetics, and both in vitro and in vivo electrophysiology (Sere et al. 2021). These techniques facilitated the characterization of neuronal activity within the LH and its relationship with the brainstem dorsal raphe nucleus, a region associated with the serotonergic system. The researchers discovered that LH neurons project to the DRN and their activity correlates with spike-wave discharges during absence seizures. This finding suggests that the LH, an area of the brain involved in autonomic regulation and heavily innervating the DRN, has a role in absence seizures and related comorbidities. It supports the hypothesis of a long-range subcortical regulation of serotonergic neuromodulation during such seizures.

However, the more combined techniques there are, the inevitably higher costs and more complex integration schemes will be faced. In summary, as the neural circuits studied become increasingly complex, the problems inherent in single techniques cannot be ignored, and the combination of multiple techniques is inevitably becoming a mainstream trend.

## Drug Therapy Targeting Neural Circuits in Epilepsy

**Fig. 3:**
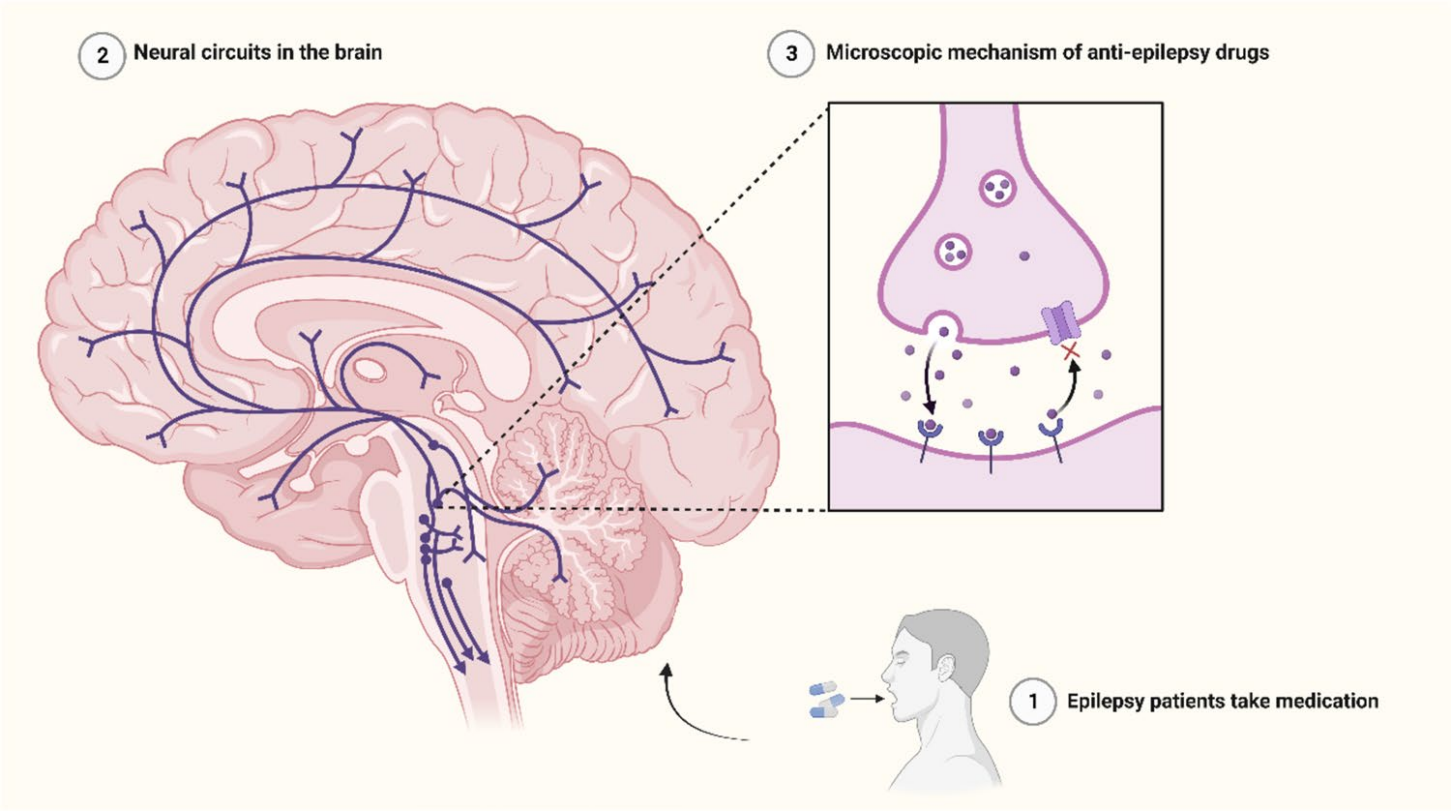
The mechanism of treating epilepsy with drugs targeting neural circuits

GABA deficiency, excessive serotonin activity, hyperactive dopamine, and glutamate excitotoxicity could enhance the occurrence of epilepsy. Neurons containing these neurotransmitters constitute the main neural circuits of epilepsy (Werner and Coveñas 2019). In recent years, more and more drugs targeting neural circuits have been developed for the treatment of epilepsy patients (Fig. 3). These drugs usually work by acting on specific neuronal cells, either exciting or inhibiting the release of synaptic vesicles, preventing excessive excitation or inhibition of nerve cells, and ultimately regulating the relevant axis to suppress epileptic seizures (Huang et al. 2024). Research on the mechanisms of actions of these drugs could help to deepen our understanding of the neural circuits in epilepsy and ultimately promote the development of related technologies (Chen et al. 2023c; Eapen et al. 2019).

Molecular-level research has always been a hot spot for the development of targeted drugs. For a long time, cannabis has been used to treat epilepsy, which was achieved by the regulation of neural circuits, despite the risk of addiction (Sugaya and Kano 2021; Russo 2017). It is known that the release of glutamate plays an important role in epilepsy (Chen et al. 2023a). Buckingham et al. found an inhibitory glutamate receptor in invertebrates, which does not exist in vertebrates (Buckingham et al. 2020). Modifying this exogenous receptor and introducing it into the human body can serve as a tool for studying neural circuits, achieving targeted therapy for epilepsy. Besides, sudden death in epilepsy is a common cause of death in patients with recurrent seizures. In response, Collard’s team conducted experiments on epileptic mice models using ghrelin analogs, and found that ghrelin analogs could significantly protect mice from respiratory arrest induced by epilepsy (Collard et al. 2022). It suggests that ghrelin analogs could be applied in the drugs to prevent respiratory failure after epileptic seizures, which is associated with the depletion of the neuropeptide in the amygdala. Additionally, studies have found that newborn hippocampal dentate granule cells might play an important role in the formation and function of epileptic neural circuits, which hold promise as a potential therapeutic target for epilepsy (Zhou et al. 2019).

Targeted drugs based on molecular mechanisms are ultimately integrated at the level of neural circuits, therefore, epilepsy treatment and research strategies should focus on the circuit level (Wang and Chen 2019). Lithium is a classic neuroprotective agent that can be effective in treating a wide range of neurological disorders. On this basis, Jiang et al. demonstrated a potential electrophysiological mechanism for lithium-induced imbalance between excitatory and inhibitory neural circuits in rat epilepsy, which involved the reactivity of hippocampal neural networks (Jiang et al. 2018a). It is known that epilepsy-regulating neuropeptides are involved in the modulation of hippocampal GABAergic neurons (Werner and Coveñas 2019). Recently, Liu’s team found that the peptide MarTX, derived from scorpions and modified, could inhibit excessive excitation of hippocampal neurons, thus holding significant promise as a potential anti-epileptic drug in the future (Liu et al. 2024).

Epilepsy has been closely linked to neural inflammation, which could be used as a basis to find specific targets (Vezzani et al. 2016). Tipton et al. first demonstrated that neuronal STAT3 might directly affect brain inflammation (Tipton et al. 2023). Inhibiting STAT3 signaling could improve the prognosis of animal models with temporal lobe epilepsy, and simultaneously prevent the pathogenic changes in the neural circuits behind epilepsy. Therefore, STAT3 could serve as an effective anti-epilepsy target. Similarly, Salazar et al. reported on the neuro-modulatory roles of forebrain inflammatory mediators, and how they could alter the balance of neural circuits behind epilepsy, ultimately affecting the epilepsy threshold (Villasana-Salazar and Vezzani 2023). This suggests that neuro-mediators and glial cells can serve as potential target cells.

In summary, research on epilepsy neural circuits can promote the development of targeted drugs, and conversely, the study of the mechanisms of action of these drugs can enhance our understanding of epilepsy neural circuits. Future research should focus on precise drugs which target neural circuits for individualized to achieve more efficient treatment.

## Conclusion and Future Perspectives

The study of neural circuits in epilepsy has witnessed a profound evolution, primarily attributable to the appearance of several cutting-edge methodologies. These techniques have substantially expanded our understanding of the fundamental neural processes and the pathophysiology related to epilepsy, facilitating the identification of particular circuits, cellular elements, and molecules involved in seizure initiation and propagation, thus establishing a solid foundation for targeted epilepsy therapies. However, every technology inevitably has certain drawbacks, and it is necessary to comprehensively consider resolution, biosafety, the range of brain areas monitored, and cost. Improving resolution and the range of brain areas monitored, while also taking biosafety into account, is the focus of future research. Due to technological leaps, research techniques for studying epileptic neural circuits have continuously broken through in the past few years, but have slowed down recently, seeming to have reached a bottleneck period. Cutting-edge research continues to push the development of technologies, while for most researchers, combining multiple technologies to overcome the disadvantages of a single technology is the most appropriate choice, although they also face the challenge of executing precise and complex plans. Combining various technologies to uncover the complex neural circuits behind epilepsy is becoming mainstream.

Additionally, investigating the neural circuits of epilepsy is significant for the development of targeted drugs. Understanding the neural circuits of epilepsy could facilitate the latest drug discovery techniques, such as targeted protein degradation, being applied in the future for epileptic drug development (Cai et al. 2023). Currently, studies are mainly at the molecular level, explaining the mechanism of action of drugs from a microscopic perspective, which helps us understand the mechanism and treatment of epilepsy. However, future research should focus more on the circuit level, considering the neural circuits of a particular brain area or even the entire nervous system. This can lead to a much deeper understanding of the specific mechanisms of epilepsy. Besides, considering the connections of neural circuits upstream and downstream can effectively reduce potential side effects and is more conducive to clinical translation. The development of drugs and the study of neural circuits should move forward together, mutually promoting each other.

Looking forward, it is important to continuously refine these methodologies and synergistically combine them to gain a more comprehensive and specific view of epileptic neural circuits. Integrative approaches, encompassing molecular, cellular, and systems-level investigations, will prove indispensable in achieving this goal. Moreover, the development of novel tools and techniques, such as advanced computational modeling and analysis, is crucial for interpreting the increasingly complex data yielded by these methodologies (Nowotny et al. 2021; Borst and Leibold 2023). Especially in recent years, with the continuous expansion of AI technology and deep learning, mathematical modeling of neural networks, such as neural mass models or small-scale models, has provided us with more accurate and convenient ways to understand the mechanisms of epilepsy (Liu and Richardson 2021). The application of these cutting-edge technologies brings hope for exploring the mechanisms of neural circuits behind epilepsy and ultimately aids in the development of anti-epileptic drugs, helping millions of patients return to a normal life.

This review also has its limitations. In the past 200 years, various techniques for studying epilepsy neural circuits have emerged, and it is inevitable that the authors missed some of them in their searching process, such as contributions from surgery and psychology (Guo et al. 2023; Gunn and Baram 2017; Mao et al. 2022). Additionally, the latest research, such as investigating neuronal connection patterns through AI algorithms and using them as the basis for functional operation, was not discussed separately due to limited literature (Gao et al. 2023; Cao et al. 2023; Zhang et al. 2021b). Undoubtedly, these technologies are also very significant for the study of epilepsy. In conclusion, the array of methods utilized to explore neural circuitry in epilepsy has resulted in significant insights into the disorder's pathophysiology. As we continue to refine and expand these techniques, the prospect of identifying new therapeutic targets and devising more effective, individualized treatment for epilepsy patients becomes ever more attainable. Through continued interdisciplinary collaboration and technological innovation, we anticipate substantial progress in the treatment and clinical management of epilepsy.

## Data Availability

Not applicable.

## Abbreviations

DREADDs: Designer receptors exclusively activated by designer drugs
DGC: Dentate gyrus cell
SST: Somatostatin
PV- INs: Parvalbumin inhibitory interneurons
S286L-TG: S286L-mutant Chrna4
iPSCs: Induced pluripotent stem cells
LH: Lateral hypothalamus
TLE: Temporal lobe epilepsy
DS: Dravet syndrome
ChR2: Channelrhodopsin-2
PV: Parvalbumin
DG: Dentate gyrus
MS: Medial septum
PIL: Posterior intralaminar thalamic nucleus
CR: Calretinin
DRN: Dorsal raphé nucleus
mTLE: Mesial temporal lobe epilepsy
